# Supplementary material for: Prevalence and psychosocial impact of atopic dermatitis in Bangladeshi children and families
Source: PLoS One. 2021 Apr 16;16(4):e0249824. doi: 10.1371/journal.pone.0249824 (PMC8051797; doi:10.1371/journal.pone.0249824)
Supplement: S1 Checklist — (DOC) [file pone.0249824.s001.doc]

STROBE Statement—Checklist of items that should be included in reports of ***cross-sectional studies***

|  | Item No | Recommendation |  | Completed |  |
| --- | --- | --- | --- | --- | --- |
| **Title and abstract** | 1 | (*a*) Indicate the study’s design with a commonly used term in the title or the abstract |  | X, see Abstract, p. 3 |  |
|  |  | (*b*) Provide in the abstract an informative and balanced summary of what was done and what was found |  | X, see Abstract, p. 3-4 |  |
| Introduction | | |  |  |  |
| Background/rationale | 2 | Explain the scientific background and rationale for the investigation being reported |  | X, see Introduction, p. 5 |  |
| Objectives | 3 | State specific objectives, including any prespecified hypotheses |  | X, see last sentence of Introduction, p. 5 |  |
| Methods | | |  |  |  |
| Study design | 4 | Present key elements of study design early in the paper |  | X, see Methods, pp. 5-6 |  |
| Setting | 5 | Describe the setting, locations, and relevant dates, including periods of recruitment, exposure, follow-up, and data collection |  | X, see Methods, pp. 5-6 |  |
| Participants | 6 | (*a*) Give the eligibility criteria, and the sources and methods of selection of participants |  | X, see Methods, pp. 5-6 |  |
| Variables | 7 | Clearly define all outcomes, exposures, predictors, potential confounders, and effect modifiers. Give diagnostic criteria, if applicable |  | X, see Methods, pp. 6-8 |  |
| Data sources/ measurement | 8* | For each variable of interest, give sources of data and details of methods of assessment (measurement). Describe comparability of assessment methods if there is more than one group |  | X, see Methods, pp. 6-8 |  |
| Bias | 9 | Describe any efforts to address potential sources of bias |  | X, see Methods, pp. 6-8 (e.g. validation) |  |
| Study size | 10 | Explain how the study size was arrived at |  | X, see Methods, pp. 5-6 |  |
| Quantitative variables | 11 | Explain how quantitative variables were handled in the analyses. If applicable, describe which groupings were chosen and why |  | X, see Data analysis, pp. 8-9 |  |
| Statistical methods | 12 | (*a*) Describe all statistical methods, including those used to control for confounding |  | X, see Data analysis, pp. 8-9 |  |
|  |  | (*b*) Describe any methods used to examine subgroups and interactions |  | N/A |  |
|  |  | (*c*) Explain how missing data were addressed |  | X, see Data analysis, p. 9 |  |
|  |  | (*d*) If applicable, describe analytical methods taking account of sampling strategy |  | N/A |  |
|  |  | (*e*) Describe any sensitivity analyses |  | N/A |  |
| Results | | |  |  |  |
| Participants | 13* | (a) Report numbers of individuals at each stage of study—eg numbers potentially eligible, examined for eligibility, confirmed eligible, included in the study, completing follow-up, and analysed |  | X, see Methods, p. 5-6 and Results, pp. 9-10 |  |
|  |  | (b) Give reasons for non-participation at each stage |  | X, see Results, p. 9 |  |
|  |  | (c) Consider use of a flow diagram |  | X |  |
| Descriptive data | 14* | (a) Give characteristics of study participants (eg demographic, clinical, social) and information on exposures and potential confounders |  | X, see Results, pp. 9-10 |  |
|  |  | (b) Indicate number of participants with missing data for each variable of interest |  | X, see Table 1 |  |
| Outcome data | 15* | Report numbers of outcome events or summary measures |  | X, see Results, pp. 9-13 |  |
| Main results | 16 | (*a*) Give unadjusted estimates and, if applicable, confounder-adjusted estimates and their precision (eg, 95% confidence interval). Make clear which confounders were adjusted for and why they were included |  | X, see Results, pp. 9-13 |  |
|  |  | (*b*) Report category boundaries when continuous variables were categorized |  | X, see Methods, pp. 7-8 and Results, pp. 9-13 |  |
|  |  | (*c*) If relevant, consider translating estimates of relative risk into absolute risk for a meaningful time period |  | N/A |  |
| Other analyses | 17 | Report other analyses done—eg analyses of subgroups and interactions, and sensitivity analyses |  | N/A |  |
| Discussion | | |  |  |  |
| Key results | 18 | Summarise key results with reference to study objectives |  | X, see Discussion, pp. 13-16 |  |
| Limitations | 19 | Discuss limitations of the study, taking into account sources of potential bias or imprecision. Discuss both direction and magnitude of any potential bias |  | X, see Limitations, pp. 16-17 |  |
| Interpretation | 20 | Give a cautious overall interpretation of results considering objectives, limitations, multiplicity of analyses, results from similar studies, and other relevant evidence |  | X, see Conclusion, p. 17 |  |
| Generalisability | 21 | Discuss the generalisability (external validity) of the study results |  | X, see Discussion, pp. 13-16 |  |
| Other information | | |  |  |  |
| Funding | 22 | Give the source of funding and the role of the funders for the present study and, if applicable, for the original study on which the present article is based |  | X, see Financial disclosure, p. 18 |  |

*Give information separately for exposed and unexposed groups.

**Note:** An Explanation and Elaboration article discusses each checklist item and gives methodological background and published examples of transparent reporting. The STROBE checklist is best used in conjunction with this article (freely available on the Web sites of PLoS Medicine at http://www.plosmedicine.org/, Annals of Internal Medicine at http://www.annals.org/, and Epidemiology at http://www.epidem.com/). Information on the STROBE Initiative is available at www.strobe-statement.org.
